# Modeling of the Potential Geographical Distribution of Three *Fritillaria* Species Under Climate Change

**DOI:** 10.3389/fpls.2021.749838

**Published:** 2022-01-10

**Authors:** Ruiping Jiang, Meng Zou, Yu Qin, Guodong Tan, Sipei Huang, Huige Quan, Jiayu Zhou, Hai Liao

**Affiliations:** School of Life Sciences and Engineering, Southwest Jiaotong University, Chengdu, China

**Keywords:** climate change, distribution, *Fritillaria*, machine learning methods, MaxEnt

## Abstract

Fritillaria species, a well-known Chinese traditional medicine for more than 2,000 years, have become rare resources due to excessive harvesting. In order to balance the economical requirement and ecological protection of *Fritillaria* species, it is necessary to determine (1) the important environmental variables that were responsible for the spatial distribution, (2) distribution change in response to climate change in the future, (3) ecological niche overlap between various *Fritillaria* species, and (4) the correlation between spatial distribution and phylogenies as well. In this study, the areas with potential ecological suitability for *Fritillaria cirrhosa*, *Fritillaria unibracteata*, and *Fritillaria przewalskii* were predicted using MaxEnt based on the current occurrence records and bioclimatic variables. The result indicated that precipitation and elevation were the most important environmental variables for the three species. Moreover, the current suitable habitats of *F. cirrhosa*, *F. unibracteata*, and *F. przewalskii* encompassed 681,951, 481,607, and 349,199 km^2^, respectively. Under the scenario of the highest concentration of greenhouse gas emission (SSP585), the whole suitable habitats of *F. cirrhosa* and *F. przewalskii* reach the maximum from 2021 to 2100, while those of *F. unibracteata* reach the maximum from 2021 to 2100 under the scenario of moderate emission (SSP370) from 2021 to 2100. The MaxEnt data were also used to predict the ecological niche overlap, and thus high overlap occurring among three *Fritillaria* species was observed. The niche overlap of three *Fritillaria* species was related to the phylogenetic analysis despite the non-significance (*P* > 0.05), indicating that spatial distribution was one of the factors that contributed to the speciation diversification. Additionally, we predicted species-specific habitats to decrease habitat competition. Overall, the information obtained in this study provided new insight into the potential distribution and ecological niche of three species for the conservation and management in the future.

## Introduction

The Fifth Assessment Report (AR5) produced by the Intergovernmental Panel on Climate Change (IPCC) stated that, with global warming, the average temperature of the earth continuously increased by 0.78°C from 2003 to 2013 compared with that from 1885 to 1990 (Stocker, 2013). The concentration of CO_2_, CH_4_, and N_2_O reached the highest level since 1200 AD. Climate change has caused substantial changes in the geographical distribution of many species. Conversely, changes in species’ ranges will affect how climate change is experienced across the landscape because surface vegetation strongly affects atmospheric properties (Fitzpatrick, 2008; Lawler, 2009; Thuiller et al., 2015). Taking the factors of population, economy, technological progress, and resource utilization into account, IPCC put forward the shared socioeconomic pathways (SSPs), in which SSP1, SSP2, SSP3, SSP4, and SSP5 represented pathway of global sustainable development, medium sustainable development, local sustainable development, unbalanced development, and routine development, respectively. SSP is an alternative and updated mode of representative concentration pathways (RCPs), providing more accurate information on climate change.

Species distribution models (SDMs), also known as ecological niche models (ENM), were applied extensively to predict potentially suitable habitats in various special and temporal ranges based on the current species distribution, richness, and environmental agents. Various SDMs, such as CLIMEX, Domain, genetic algorithm for rule-set production (GARP), Bayesian network, and maximum entropy (MaxEnt), have been used to evaluate the ecological requirements, ecological responses, and distribution areas (Midgley and Thuiller, 2005). Among these modeling approaches, MaxEnt is widely used since it performs better with small sample sizes compared with other modeling methods (Ma and Sun, 2018; Perkins-Taylor and Frey, 2020). For example, Ma and Sun (2018) analyzed the distribution of *Stipa purpurea* across the Tibet using MaxEnt model, and therefore provided evidence for establishing reasonable management practices. Zhang et al. (2018) predicted the potential geographical distribution of two peony species under a change of CO_2_ concentration. Abou-Shaara et al. (2021) speculated on the possible invasion of *Oplostomus fuligineus* into North Africa and South Europe from 2050 to 2070.

The genus *Fritillaria* has attracted much attention because of its commercial value, partly as ornamental plants, but mostly as medicinal plants in traditional Chinese medicine (TCM). The bulbs of *Fritillaria* species, called as Beimu, were used by humans for >2,000 years to treat cough and phlegm. According to the Chinese Pharmacopeia 2015, the bulbs of *F. cirrhosa*, *F. unibracteata*, *F. przewalskii*, *F. delavayi*, *F. unibracteata var wabuensis*, and *F. taipaiensis* were used as Chuan-Bei-mu in Chinese medicine markets (Chen et al., 2020). In recent years, high market prices of Chuan-Bei-mu had led to excessive harvesting and unsustainable decline in plant distribution of wild *Fritillaria* populations. Moreover, the *Fritillaria* bulbus became smaller and deeper in response to heavy collecting (Li X. et al., 2017). At present, *F. cirrhosa*, *F. unibracteata*, and *F. prewalskii*, distributed in the alpine areas of the Himalayan–Hengduan Mountains, have been classified as endangered species and those wild resources were rarely found in China. During the past decades, the Himalayan–Hengduan Mountains experienced an increase in average temperature (0.03–0.05°C year^–1^) (Salick et al., 2019). The alpine plants in the Himalayan–Hengduan Mountains region were expected to be sensitive to anthropogenic climate change since their biota was generally cold-adapted (Wang et al., 2020). However, the adaptive rate of species may be slow compared to the rate of climate change (Quintero and Wiens, 2013), and may lead to range contractions (Giménez-Benavides et al., 2011) and/or local extinctions (Wiens, 2016). Therefore, in order to develop a scientific conservation strategy, conservative approaches, as well as artificial cultivation, it is necessary to predict the geographical distribution of *Fritillaria* species, especially those rare species. To date, there were at least four factors that contributed to the geographical distribution of plants. First of all, the geographical distribution of plants was undoubtedly sensitive to environmental factors (Sosef et al., 2017). For instance, Gao et al. (2019) showed that the reproductive success of *F. delavayi* was critically affected by the temperature and ultraviolet-B/C intensity, and as a result, *F. delavayi* was distributed in a narrow altitude area. Thus, both detecting the dominant environmental factors involved in the geographical distribution of various *Fritillaria* species and identifying ecologically suitable areas of various *Fritillaria* species are critical issues for designing conservation and cultivation plans for the future. Second, although the most commonly documented response of plants to climate change were distributional range shifts to higher latitudes and/or elevations (Parmesan and Yohe, 2003; Zhang et al., 2018), it was also necessary to predict how various developmental models would determine the spatial extent of their suitable habitat. Third, the ecological niche overlaps between species with close relationships might produce habitat competition to some extent (Eyres et al., 2020), and thus predicting the spatial distribution of various *Fritillaria* species might find species-specific habitat to decrease competition. Fourth, the distribution of a species is an expression of its evolutionary history and its ecology (Soberón and Peterson, 2005; Villegas et al., 2021). Prediction of geographic distributions is not only useful for identifying environmental factors that may limit the range of species, but also is useful for inferring patterns of speciation. Therefore, the correlation between spatial distribution and phylogenies received much attention in recent decades, despite the fact that it remained elusive (Savolainen et al., 2013).

In this study, we extensively collected the current distributing records of *F. cirrhosa*, *F. unibracteata*, and *F. przewalskii*. We further used MaxEnt and GARP modeling to evaluate the properties of habitat distribution and environmental variables shaping habitat. We tested the overlapping distribution of various original species. The objectives of this study were to (1) identify the most important environmental variables that contributed to the ranges of these three species, (2) examine the spatial changes in the size of suitable habitat under various developmental models in the future; (3) evaluate the overlap extent of three species and discover species-specific suitable habitat for each species; and (4) evaluate the correlation between spatial distribution and phylogenies.

## Materials and Methods

**FIGURE 1:**
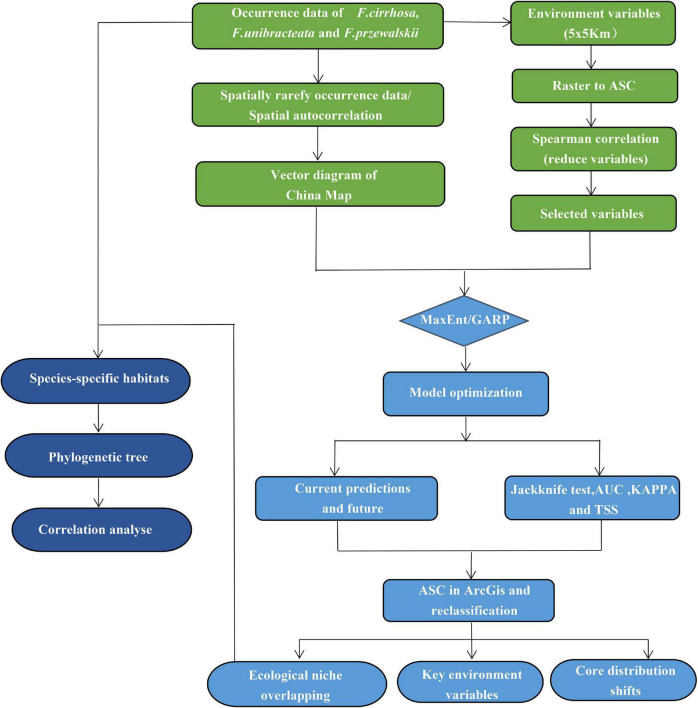
Processing methods in the roadmap of this study.

The full roadmap on which analyses were based is summarized in Figure 1.

### Occurrence Data and Processing

**FIGURE 2:**
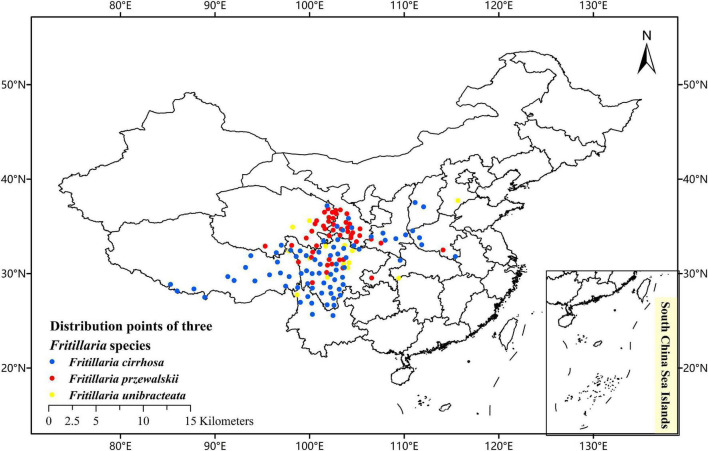
Current occurrence of *Fritillaria cirrhosa*, *Fritillaria unibracteata*, and *Fritillaria przewalskii* in China.

To collect the occurrence records of *F. cirrhos*a, *F. unibracteata*, and *F. przewalskii*, respectively, we conducted an extensive literature search using online databases and referred to the Chinese Virtual Herbarium database (CVH)^1^ and the plant photo bank of China (PPBC),^2^ global biodiversity information facility (GBIF),^3^ and field investigation as well. Then, records lacking latitude and longitude were georeferenced using Google maps module that is included in the Chinese Satellite Map.^4^ Using the above resources, the distributional localities of *F. cirrhosa*, *F. unibracteata*, and *F. przewalskii* were added to a database. After deleting the duplicated records and filtering spatially by Buffer analysis, only one point was left within each grid cell (buffer radius is 20 km). Furthermore, to avoid sampling bias, spatial autocorrelation was measured by calculating Moran‘s *I* using GeoDa v1.14 software (Wu et al., 2021). Finally, the remaining records were used for constructing the models. With the help of ArcGIS 10.2, the sighting point map was obtained (Figure 2).

### Environment Variables

Initially, 22 environmental variables (Supplementary Table 1) that might influence the distribution of *F. unibracteata*, *F. cirrhosa*, and *F. przewalskii* were selected to model the current species distribution patterns. These variables included 19 bioclimatic variables that described annual and seasonal temperature and rainfall trends (WorldClim version 2.0, Fick and Hijmans, 2017) and three topographic variables (slope, altitude, and aspect extracted from elevation data through the spatial analysis module in ArcMap 10.2.). The 19 WorldClim variables have been broadly used for generating ENMs, including successful use with plants in the Himalayan–Hengduan Mountains (Ma and Sun, 2018; Zheng et al., 2021). These three topographic variables have been indicated to play a role in the distribution and density of *Fritillaria* species (Lakey and Dorji, 2016).

For future climate scenarios, we used BCC-CSM1.1 climate change modeling data underlying the shared socioeconomic pathways SSP126, SSP245, SSP370, and SSP585 scenarios released by the CMIP6 and IPCC Assessment Report 6 (AR6). BCC-CSM 1.1 is among the most-used models currently available for simulating the global climate response to increasing greenhouse gas concentration. Our four selected future climate data sets, including 2021–2040, 2041–2060, 2061–2080, and 2081–2100, were downloaded from the World Climate Database (Abou-Shaara et al., 2021) with a spatial resolution of 2.5 arc-min. All the environmental variables were in raster format and were prepared in ArcGIS 10.2 to align in the geographic space using a WGS84 datum system with default parameters.

As the high collinearity of 22 environmental variables may lead to the model overfitting, the Spearman correlation analysis was used to test multicollinearity among these variables. Before the correlation analysis, the environmental variables with a contribution rate <0.3 were removed. According to the present distribution points of *Fritillaria*, the associated environmental variables were extracted and with those values, a correlation matrix in SPSS 20 software was performed. Then, following the methods of Jiang (2017) and Wang et al. (2018), we identified environmental variables that had a coefficient of correlation >0.8 with other environmental variables and eliminated the one with a smaller contribution rate (Supplementary Table 2). Thus, the remaining environmental variables were used in the further prediction of models (Feng et al., 2019).

### MaxEnt and GARP Modeling

We used the MaxEnt (MaxEnt version 10.2) and GARP models. For each species, various training/testing sets (80/20, 75/25, and 70/30) were used for validating the models. The algorithm runs 500 iterations of these processes. We selected test samples by the bootstrap method. The Jackknife method was checked in the setting of environmental parameters and the response curve of environmental variables was created. The remaining settings were left at the default setting; finally, the test was repeated 10 times. The outputs were transformed into raster format using the ArcMap tool in ArcGIS software for further analysis.

The effectiveness of MaxEnt and GARP models was evaluated by three standard performance criteria, including area under the curve (AUC) of the receiver operating characteristic (ROC) curve, true skill statistics (TSS), and Kappa values. AUC of the ROC curve is the threshold-independent measure of model accuracy, which juxtaposes correct and incorrect predictions over a range of thresholds (Zhu et al., 2012). The AUC value was calculated as the area enclosed by the ROC curve and generally ranges between 0 and 1. A larger AUC value indicates better model precision. The AUC values could be grouped as failing (AUC ≤ 0.6), poor (0.6–0.7), moderate (0.7–0.8), good (0.8–0.9), and excellent (0.9–1). TSS scores range from -1 to 1, where +1 indicates a perfect ability to distinguish suitable from unsuitable habitat, while values of zero or less indicate a performance no better than random (Assefa et al., 2021). Kappa (Schatz et al., 2017; Fernández and Morales, 2019) measures how closely model predictions match the truth, controlling for random accuracy by estimating the expected accuracy. Kappa values range from -1 to 1, where 1 indicates 100% agreement, and -1 indicates 100% disagreement.

We imported prediction results into ArcGIS and then drew global maps of ecologically appropriate zones for three *Fritillaria* species, respectively. During the ArcGIS mapping, artificial grading was used to classify different grades based on their ecological similarity. The final potential species distribution map had a range of values from 0 to 1 which were regrouped into four classes of suitable habitats, including highly suitable habitat (0.75–1), moderately suitable habitat (0.5–0.75), low suitable habitat (0.25–0.5), and not suitable habitat (0–0.25) (Ren et al., 2020). These three levels of suitable habitats (25–100%) indicated that species have a probability of survival in these areas (Swets, 1998). The suitable ranges of the environmental variables were defined as those ranges where species inhabited survival areas with low, moderate, and high suitability (≥0.25) (Zhang et al., 2018). The range of appropriate eco-factor values was derived from the response curves of the MaxEnt model results.

### Prediction on Niche Breadth and Niche Overlap of Three *Fritillaria* Species

The niche breadth in geographical and environmental space was calculated for each species as the mean Levins’ B1 (inverse concentration) and B2 (uncertainty) values (Levins, 1968) from habitat suitability maps of each species with the software ENMTools v1.3 (Warren et al., 2008). Levins’ B1 and B2 values range from 0 to 1, with values closer to 0 representing narrow niche breadth, and values closer to 1 representing wide niche breadth. The ENMTools package was further used to evaluate niche overlap based on the values of two indexes, Schoener’s *D* (Schoener, 1968) and Hellinger’s *I* (Warren et al., 2008). Schoener’s *D* and Hellinger’s *I* values range from 0 to 1, with values closer to 0 representing a small degree of niche overlap, while values closer to 1 representing a high degree of niche overlap.

To strengthen our speculation, the overlapping degree between highly and moderately suitable habitats of paired species is defined as follows, which is the proportion of the records in the overlapping region divided by their union.


$$Overlappingdegree=\frac{A_{overlap}+B_{overlap}}{A_{total}+B_{total}}$$

The A_overlap_ and B_overlap_ represent the number of records of species A and species B in the overlapping regions, respectively. While A_total_ and B_total_ are the numbers of all existing records that are related to the respective individual species.

### DNA Sequences and Phylogenetic Analysis

Internal transcribed spacer 1 (ITS1) and internal transcribed spacer 2 (ITS2) are the internal regions between 18S and 5.8S, 5.8S, and 28S, respectively. Both have been widely used as molecular markers to explore phylogenetic relationships (Cheng et al., 2016; Khan et al., 2019). While, as a promising method, phylogenetic analysis based on the Chloroplast genome (CP) was also applied in our research. We then searched the GenBank database for DNA sequences of ITS1, ITS2, and CP data, some of which were obtained by our laboratory previously (Zheng et al., 2019). Our strategy included (1) marker sequences available for all the species; (2) sequences that could be linked for all species; (3) sequences contributing to extensive gaps in the conserved regions of the alignment were discarded; and (4) sequences that could be assigned to a set of geographic coordinates.

The ITS1+ITS2, ITS1, ITS2, and CP trees were constructed using a maximum likelihood using the MEGA 7.0 (Deng et al., 2018). To obtain statistical support on the resulting clades, a bootstrap analysis with 1,000 replicates was performed. The evolutionary distance was defined as the number of nucleotide substitutions occurring between various sequences based on the Tamura–Nei model (Tamura and Nei, 1993). In the maximum likelihood tree, the pairwise distance was exported to calculate the evolutionary distance (Kumar et al., 2018), which was used for the further correlation analysis.

### Statistical Methods

A linear trend line has been drawn through the evolutionary distances and the occurrence records in the overlapping area. The R^2^ value indicates how well data fit the line. To test the statistical significance of the correlation results, a Pearson Product Moment Correlation test was performed using SigmaStat v3.5 software. A correlation coefficient (ρ) positive and a *p*-value lower than 0.05 means that the two variables tend to increase in a concerted manner (Santamaría et al., 2014).

## Results

### Model Performance and Contribution of Environmental Variables

In the initial step, 112, 51, and 73 native occurrence records of *F. cirrhosa*, *F. unibracteata*, and *F. przewalskii*, respectively, were obtained. After filtering data, the records of *F. cirrhosa*, *F. unibraceate*, and *F. przewalskii* were decreased to 77, 45, and 65, respectively (Supplementary Table 3). The corresponding Moran *I* of three *Fritillaria* species were 0.0621465, -0.01864, and 0.0016039, respectively. The procedure greatly reduced sampling bias and spatial autocorrelation, resulting in evenly distributed occurrence points across space, and thus the remaining records could be used for further model construction.

**FIGURE 3:**
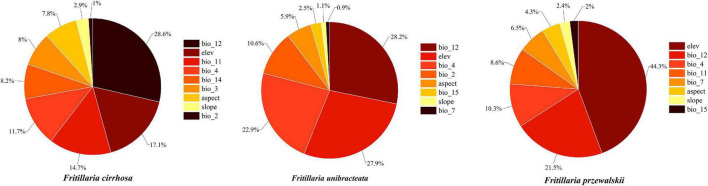
The contribution rate of each environment variable to the modeling of MaxEnt.

With the given training and testing sets (80/20, 75/25, and 70/30), the MaxEnt model performed better than the GARP model by evaluation metrics (AUC, KAPPA, and TSS). When training/testing set was 75/25, *F. unibracteata* (AUC = 0.972 (SD = 0.006), KAPPA = 0.913, and TSS = 0.890) and *F. przewalskii* (AUC = 0.981 (SD = 0.006), KAPPA = 0.871, and TSS = 0.913) had better performance, while 70/30 set was more suitable for *F. cirrhosa* [AUC = 0.969 (SD = 0.006), KAPPA = 0.731, and TSS = 0.730] (Supplementary Table 4). Therefore, MaxEnt was used as the final model for three *Fritillaria* species, in which *F. unibracteata* and *F. przewalskii* employed 75/25 set, while *F. cirrhosa* employed 70/30 set. The internal jackknife test in the MaxEnt software package verified the importance of various environmental variables on the three *Fritillaria* species, respectively (Figure 3). When used independently, Bio12 (23.7% of contribution), Bio11 (17.7% of contribution), Elev (16.5% of contribution), and Bio4 (15.5% of contribution) provided the greatest contributions to the distribution model for *F. cirrhosa* relative to other variables. The cumulative contributions of these four factors reached values as high as 73.4%, indicating that these variables contained more useful information than the other variables. Bio12 (28.2% of contribution), Elev (27.9% of contribution), Bio4 (22.9% of contribution), and Bio2 (10.6% of contribution) made the highest contributions to the distribution model of *F. unibracteata*. The cumulative contributions of the variables reached values as high as 89.6%. Furthermore, the environmental variables that provided the greatest contributions to *F. przewalski*i were Elev (44.3% of contribution), Bio12 (21.5% of contribution), and Bio4 (10.3% of contribution). Therefore, the important environmental factors that influenced the suitable habitat were identified as Bio12, Bio11, Elev, and Bio4 for *F. cirrhosa*, Bio12, Elev, Bio4, and Bio2 for *F. unibracteata*, and Elev, Bio12, and Bio4 for *F. przewalskii*, respectively.

**FIGURE 4:**
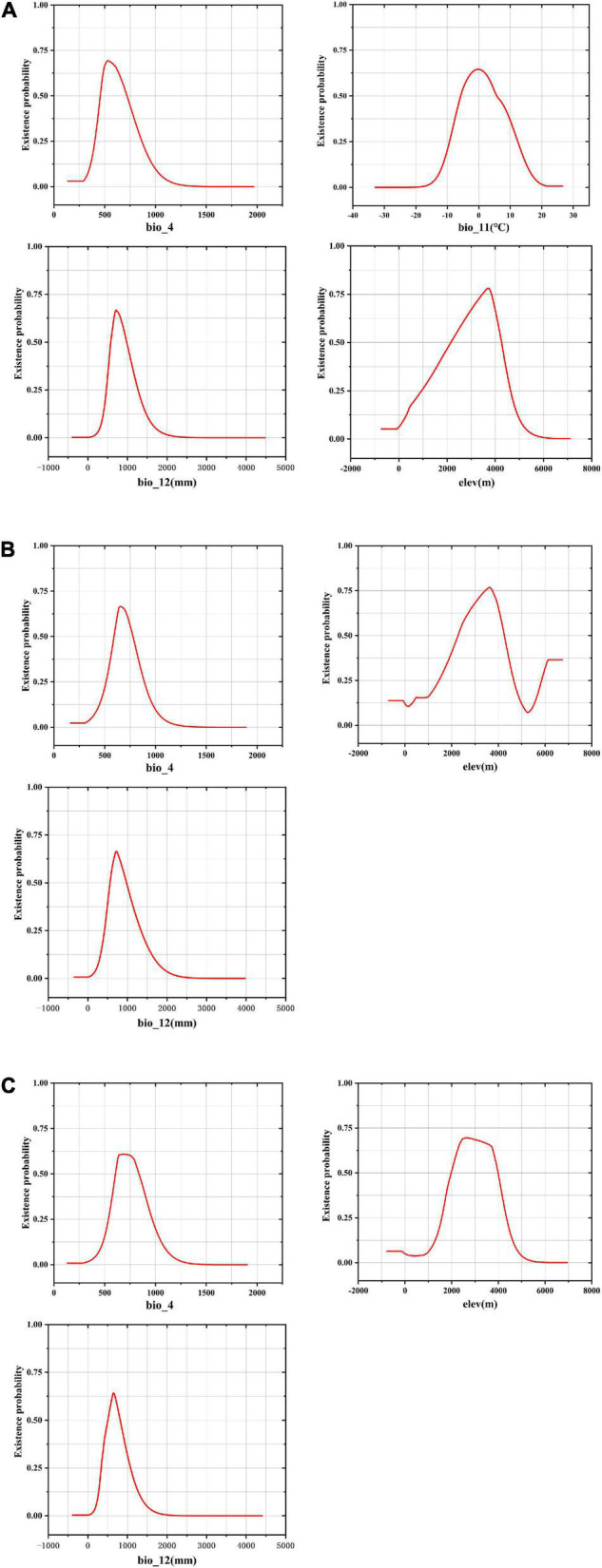
Response curves for dominant environmental variables in the species distribution model for *Fritillaria cirrhosa* **(A)**, *Fritillaria unibracteata* **(B)**, and *Fritillaria przewalskii* **(C)**.

The response curve of environmental variables takes the specific value of environmental variables as Abscissa and the existence probability of species as ordinate, which reflected the suitable range of environmental variables in the study area (Figure 4). The suitable ranges of the environmental variables were defined as those ranges where three *Fritillaria* species inhabited survival areas with low, moderate, and high suitability (≥0.25) (Zhang et al., 2018).

In *F. cirrhosa*, the suitable ranges of Bio12, Elev, Bio4, and Bio11 (see definitions in Table S1) were 468.68–1263.40 mm, 949.40–4631.11 m, 403.91–865.23, and -9.28 to 12.33°C, respectively. When Bio12 exceeded 468.68 mm, the probability for *F. cirrhosa* increased gradually, and reached the maximum at 707.59 mm with a probability of existence as high as 0.67. When Elev exceeded 949.40 m, the survival probability of *F. cirrhosa* first increased and then decreased, and reached 3716.54 m with the highest probability of existence as 0.78. When the Bio4 reached 532.05, *F. cirrhosa* obtained the highest probability of existence as 0.69. Finally, it achieved the top probability of existence as 0.65 once Bio11 reached -0.17°C (Figure 4A). In *F. unibracteata*, the optimum values of Bio12, Elev, and Bio4 ranged from 421.41 to 1338.27 mm, 1453.41 to 4653.12 m, and 513.42 to 888.86, respectively (Figure 4B). Furthermore, in *F. przewalskii*, the optimum ranges of Bio12, Elev, and Bio4 were from 335.98 to 1073.34 mm, from 1576.31 to 4342.94 m, from 537.14 to 967.75, respectively (Figure 4C). The existence probability of three *Fritillaria* species increased at first and then decreased with the increase of environmental factors. Table 1 shows the suitable range of environmental variables corresponding to the three *Fritillaria* species.

**TABLE 1:** Suitable range and optimum environmental variables of three *Fritillaria* species.

Species	Environmental variables	Suitable range	Optimum	Maximum probability of existence
*F. cirrhosa*	Bio12 (mm)	468.68–1263.40	707.59	0.67
	Elev (m)	949.40–4631.11	3716.54	0.78
	Bio4	403.91–865.23	532.05	0.69
	Bio11 (°C)	−9.28 to 12.33	–0.17	0.65
*F. unibracteata*	Bio12 (mm)	421.41–1338.27	715.50	0.66
	Elev (m)	1453.41–4653.12	3618.80	0.77
	Bio4	513.42–888.86	657.02	0.67
*F. przewalskii*	Bio12 (mm)	335.98–1073.34	647.21	0.64
	Elev (m)	1576.31–4342.94	2596.41	0.70
	Bio4	537.14–967.75	691.31	0.61

### The Currently Suitable Habitats of Three *Fritillaria* Species

**FIGURE 5:**
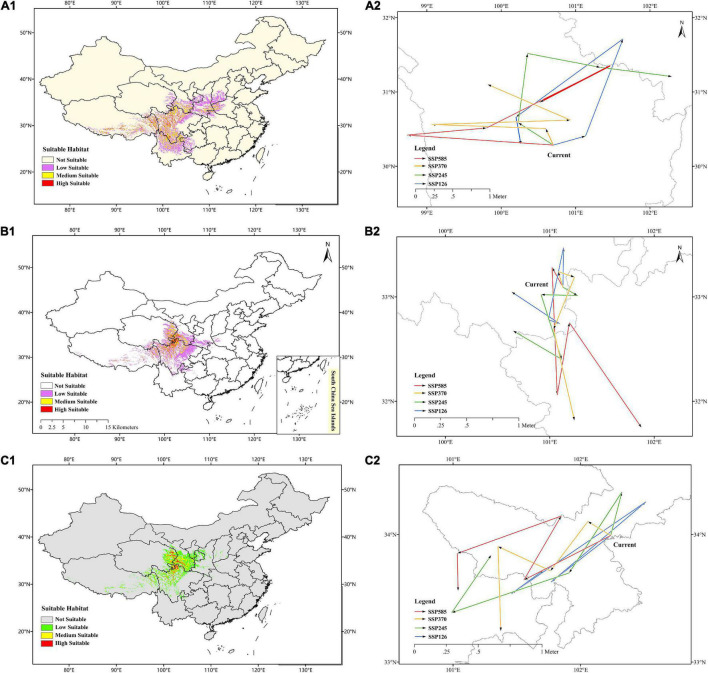
Predicted current distribution model and the core distributional shifts under different climate scenarios/year for three *Fritillaria* species. **(A1–C1)** Represented predicted current distribution for *Fritillaria cirrhosa*, *Fritillaria unibracteata* and *Fritillaria przewalskii*, respectively. **(A2–C2)** Represented core distributional shifts under different climate scenarios/year for *Fritillaria cirrhosa*, *Fritillaria unibracteata* and *Fritillaria przewalskii*, respectively.

The prediction of current habitats for *F. cirrhosa* showed that the highly suitable habitat occurred in Sichuan, Tibet, Yunnan, Gansu, Shanxi, Henan, Qinghai, Guizhou, and Hubei provinces. The evaluated moderately suitable habitat distributed in Shanxi, Ningxia, Chongqing, and Hebei. The current areas of suitable habitats for *F. cirrhosa* were up to 0.68 million km^2^, accounting for 7.10% of the total area of China (Figure 5A1).

In *F. unibracteata*, the evaluated highly suitable habitat was located in Sichuan, Qinghai, Gansu, and Tibet. The evaluated areas of moderately suitable habitat included Sichuan, Tibet, Qinghai, Gansu, Shaanxi, Henan, Hubei, and Yunnan. The current areas of suitable habitat for *F. unibracteata* encompassed 0.48 million km^2^ (Figure 5B1). As for *F. przewalskii*, the highly suitable habitat occurred in Gansu, Qinghai, Sichuan, and Tibet and the moderately suitable habitat distributed around the highly suitable growth area. The total area suitable habitat for *F. przewalskii* was 0.35 million km^2^, accounting for 3.64% of the total area of China (Figure 5C1).

### Future Changes in Suitable Habitat Area

The centroid of the current habitat of *F. cirrhosa* was located at the site of 100°699′E and 30°275′N in south-west Sichuan province. The centroid of the suitable area shifted to 101°130′E, 30°399′N, 101°639′E, 31°704′N, 100°200′E, 30°656′N, 100°264′E, 30°3′N under SSP126 from 2021 to 2100. Under SSP245, the centroid of the future suitable area moved to 100°235′E, 30°584′N, 100°35′E, 31°507′N, 101°315′E, 31°331′N, 102°292′E, 31°302′N from 2021 to 2100. Under SSP370, the centroid of the future suitable area changed to 100°577′E, 30°486′N, 99°121′E, 30°558′N, 100°917′E, 30°611′N, 99°827′E, 31°089′N. Moreover, on the condition of SSP585, the centroid of the suitable area shifted to 98°741′E, 30°406′N, 99°782′E, 30°505′N, 101°460′E, 31°334′N, 100°540′E, 30°859′N. Overall, the core distribution of *F. cirrhosa* shifted toward the north-east under four scenarios (Figure 5A2). The elevation of the centroid under four scenarios (4,143–4,601 m) was higher than that of the current centroid (3,907 m).

In *F. unibracteata*, the centroid of the current habitat was located at the position of 101°124′E, 33°092′N in north-west Sichuan province. The centroid of the suitable area shifted to 101°132′E, 33°468′N, 100°991′E, 32°789′N, 101°087′E, 32°745′N, 100°645′E, 33°041′N from 2021 to 2100 under SSP126. Under SSP245, the centroid of the future suitable area moved to 101°257′E, 33°018′N, 100°918′E, 33°022′N, 101°114′E, 32°407′N, 100°653′E, 32°673′N from 2021 to 2100. Under SSP370, the centroid of the future suitable area changed to 101°081′E, 33°238′N, 101°236′E, 33°189′N, 101°038′E, 32°691′N, 101°238′E, 31°838′N from 2021 to 2100. Finally, under the condition of SSP585, the centroid of the suitable area shifted to 101°150′E, 33°078′N, 101°078′E, 32°070′N, 101°192′E, 32°732′N, 101°877′E, 31°742′N from 2021 to 2100. Overall, the core distribution of *F. unibracteata* shifted toward the south under four scenarios (Figure 5B2).

Additionally, in *F. przewalskii*, the centroid of the current habitat was located at the position of 102°236′E, 33°99′N in north-west Sichuan province. The centroid of the suitable area shifted to 101°769′E, 33°627′N, 102°511′E, 34°256′N, 101°459′E, 33°536′N, 101°708′E, 33°719′N from 2021 to 2100 under SSP126. Under SSP245, the centroid of the future suitable area moved to 102°328′E, 34°331′N, 101°911′E, 33°697′N, 100°996′E, 33°39′N, 101°299′E, 33°834′N from 2021 to 2100. Under SSP370, the centroid of the future suitable area changed to 102°059′E, 34°102′N, 101°766′E, 33°711′N, 101°355′E, 33°904′N, 101°377′E, 33°246′N from 2021 to 2100. Finally, under the condition of SSP585, the centroid of the suitable area shifted to 101°559′E, 33°647′N, 101°85′E, 34°141′N, 101°036′E, 33°852′N, 101°039′E, 33°564′N from 2021 to 2100. Generally, the core distribution of *F. przewalskii* shifted slightly toward the south-west under four scenarios (Figure 5C2). The elevation of the centroid under four scenarios (3,503–4,284 m) also increased compared with that of the current centroid (3,425 m).

**FIGURE 6:**
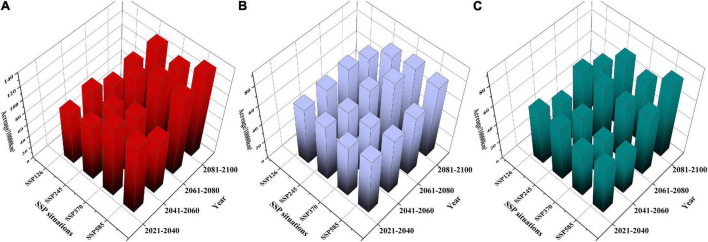
The suitable areas of three *Fritillaria* species in China under future climate conditions. **(A)** *F. cirrhosa*, **(B)** *F. unibracteata*, and **(C)** *F. przewalskii.*

As shown in Supplementary Table 5, in *F. cirrhosa* (Figure 6A), the areas of highly and moderately suitable habitats, as well as suitable habitats (including high, moderate, and low suitable habitats) increased in a wave-like change under the SSP126 scenario. The maximum area of highly and moderately suitable habitats appeared in 2041–2060 with 0.48 and 2.32%, respectively. Under the SSP245 scenario, the highly and moderately suitable habitats, as well as the suitable habitat continuously increased from 2020 to 2100. Both the maximum area of highly and moderately suitable habitats appeared in 2081–2100 with 0.97 and 4.12%, respectively. Moreover, under the SSP370 scenario, the highly and moderately suitable habitats showed a similar increasing tendency as SSP126. The highly and moderately suitable habitats reached the maximum at 2061–2080 period with 1.39 and 4.01%, respectively. Finally, under the SSP585 scenario, the highly and suitable habitats as well as suitable habitat showed similar tendencies as those under the SSP126 scenario from 2020 to 2100. The highly and moderately suitable habitats reached the maximum at 2081–2100 with 1.53% and at 2061–2080 with 3.95%, respectively. Overall, SSP585 was the optimum scenario for *F. cirrhosa*, since the highly and totally suitable habitats reached the maximum areas during 2021–2100 at 146,758 and 133,884.2 km^2^, respectively.

In *F. unibracteata* (Figure 6B), the areas of highly and moderately suitable habitats as well as suitable habitat increased first and then declined slightly (but higher than that of the current corresponding habitat) under the SSP126 scenario. The maximum area of the highly and moderately suitable habitats appeared in 2061–2080 with 0.36 and 1.66%, respectively. Under the SSP245 scenario, the highly and moderately as well as totally suitable habitat increased in a wave-like change. The maximum area of highly and moderately suitable habitats appeared in 2041–2060 with 0.46% and in 2081–2100 with 1.61%, respectively. Furthermore, under the SSP370 scenario, the highly and moderately, as well as totally suitable habitat showed a similar tendency as those under the SSP245 scenario. The highly and moderately suitable habitats reached the maximum at 2081–2100 with 0.54% and at 2061–2080 with 1.96%, respectively. Finally, under the SSP585 scenario, the highly and moderately as well as the totally suitable habitat increased continuously. Both highly and moderately suitable habitats reached the maximum at 2081–2100 with 0.36 and 2.08%, respectively. Overall, SSP370 was the optimum scenario for *F. unibracteata*, since the highly and totally suitable habitats reached the maximum areas during 2021–2100 at 517.39 and 831.501 km^2^, respectively.

In *F. przewalskii* (Figure 6C), the areas of highly and moderately as well as totally suitable habitat increased in a wave-like change under SSP126, SSP245, SSP370, and SSP585 scenario, respectively. Under the SSP126 scenario, both the maximum area of highly and moderately suitable habitats appeared in 2061–2080 with 0.65 and 1.66%, respectively. Under the SSP245 scenario, the maximum area of both highly and moderately suitable habitats appeared in 2080–2100 with 0.89 and 2.24%, respectively. Additionally, under the SSP370 scenario, the highly and moderately suitable habitats reached the maximum at 2061–2080 with 0.84% and 2081–2100 with 2.00%, respectively. Finally, under the SSP585 scenario, the highly and moderately suitable habitats reached the maximum at 2081–2100 with 1.10 and 2.43%, respectively. SSP585 was the optimum scenario for *F. przewalskii* since the highly, moderately, and suitable habitats reached the maximum areas during 2021–2100 at 106,043, 232,835, and 784,581 km^2^, respectively.

### Ecological Niche Overlapping of Three *Fritillaria* Species

**FIGURE 7:**
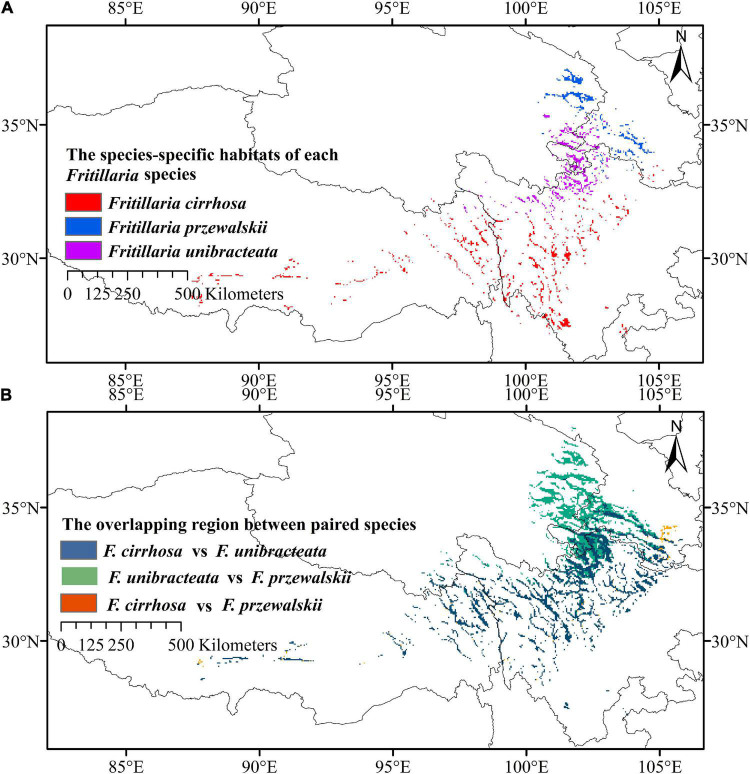
Distribution patterns of three *Fritillaria* species. **(A)** Species-specific high suitable habitats of *Fritillaria cirrhosa*, *Fritillaria unibracteata*, and *Fritillaria przewalskii*. **(B)** Overlapping region of high and medium suitable habitats between paired species.

We carried out a prediction on the ecological niche overlap of three *Fritillaria* species based on the distribution maps (Figure 2). As a result, *F. unibracteata* showed the broadest niche (indicated by the highest value of B1 and B2: 0.187 and 0.908, respectively), while the *F. cirrhosa* showed a narrower niche (B1 and B2: 0.155 and 0.886, respectively) and *F. przewalskii* showed the narrowest (B1 and B2: 0.112 and 0.874). The most similar niches were of *F. unibracteata* and *F. przewalskii*, which is indicated by the highest values of niche overlap (*I*: 0.927, *D*: 0.723). *Fritillaria cirrhosa* was more similar to *F. unibracteata* (*I*: 0.896, *D*: 0.659) than to *F. przewalskii* (I: 0.879, D: 0.619). Then, based on the statistics and calculations, the overlapping degree in highly and moderately suitable habitats of *F. cirrhosa* and *F. unibracteata* was 34.43%, those of *F. cirrhosa* and *F. przewalskii* was 26.06% and those of *F. unibracteata* and *F. przewalskii* was 50.00% (Figure 7B). The results coincided with the results of prediction by ENMTools that *F. unibracteata* and *F. przewalskii* shared the highest niche overlap.

After predicting the overlapping and specific suitable habitats of the three *Fritillaria* species, we found that the densely overlapping highly and moderately suitable habitats were mainly distributed in the Himalayan–Hengduan Mountains region, and mainly concentrated in Sichuan, Tibet, Qinghai, Gansu, and Shanxi provinces (Figure 7B). The specific highly suitable habitat of *F. cirrhosa* was distributed at southwest Sichuan, north Yunnan, east Tibet, and some were distributed at Shaanxi, Henan, and Hubei. The specific highly suitable habitat of *F. unibracteata* was mainly concentrated in north Sichuan, south Qinghai, and west Gansu. The specific highly suitable habitat of *F. przewalskii* was mainly concentrated in Gansu, Qinghai, and Sichuan province as shown in Figure 7A.

**FIGURE 8:**
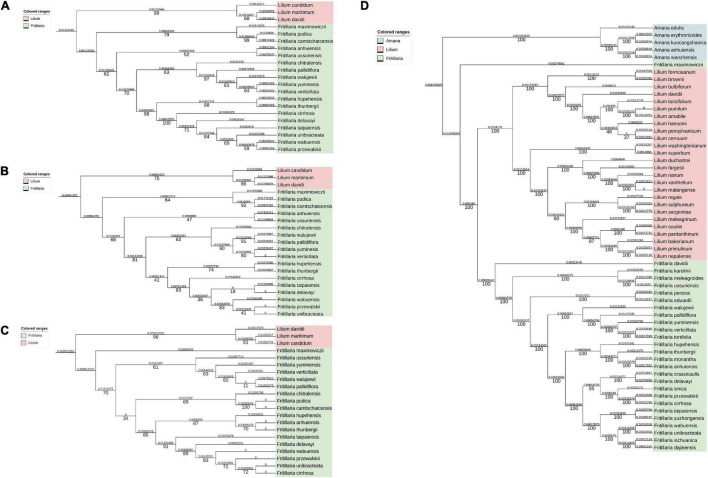
Phylogenetic trees based on ITS1, ITS2, ITS1+ITS2, and CP genome. The pairwise distance (above the branches) and bootstrap value (under the branches) were listed. **(A)** Phylogenetic trees based on ITS1 sequences. **(B)** phylogenetic trees based on ITS2 sequences; **(C)** phylogenetic trees based on ITS1+ITS2 sequences; **(D)** phylogenetic trees based on CP genomes.

To study the correlation between the overlapping extent and the evolutionary distance between different *Fritillaria* species, ITS1 (Figure 8A), ITS2 (Figure 8B), ITS1+ITS2 (Figure 8C), and CP (Figure 8D) were obtained, and further the phylogenetic trees were constructed (Figure 8) and the corresponding evolutionary distance was calculated (Supplementary Table 6). As shown in Table 2, the correlation between evolutionary distances based on ITS2 and CP, and overlapping extent showed weak accuracy (no correlation, *R*^2^ = 0.289) with no significant correlation (*P* >> 0.05), whereas that of ITS1 obtained medium accuracy (*R*^2^ = 0.766) but high *P*-value. The evolutionary distances based on ITS1+ITS2 and overlapping extent showed a positive correlation with relatively higher accuracy (*R*^2^ = 0.93) and lower *P*-value (*P* = 0.12).

**TABLE 2:** The correlation between distribution pattern and phylogenetic relationship.

DNA sequences	R^2^	*P*-value	Evolutionary distance		
			*F. cirrhosa* vs. *F. unibracteata* (62.76%)	*F. cirrhosa* vs. *F. przewalskii* (50.91%)	*F. unibracteata* vs. *F. przewalskii* (67.54%)
ITS1+ITS2	0.93	0.12	0.00991	0.0116	0.00164
ITS1	0.766	0.222	0.0109	0.0109	0
ITS2	No correlation	No correlation	0	0	0
CP genome	0.289	0.409	0.00217	0.000195	0.00231

*The overlapping extent between paired species was listed in the bracket.*

## Discussion

As classical method, SDMs utilized current data to build a correlative model that met ecological requirements of species and thus predicted the relative habitat suitability in the future (Warren and Seifert, 2011). To obtain reliable prediction, it was necessary to improve the quality and accuracy of current data rigorously. During the initial process of collecting records, several records were excluded due to highly spatial autocorrelation. This procedure would avoid sample bias and was suggested by Zhu et al. (2012) and Wu et al. (2021). Second, the correlation analysis and reasonable selection of environmental variables would reduce the complexity of machine learning models and increase the researching sense. Similar studies have been reported extensively (Wang et al., 2018; Feng et al., 2019; Zurell et al., 2020). Meanwhile, various SDMs, parameterization, and evaluation index would provide comprehensive reliability of models. Especially, although most literatures employed 75% data as training set and 25% data as testing set (Wang et al., 2018; Zhang et al., 2018; Ren et al., 2020), our result indicated that the proportion of training/testing should be considered. Prediction of GARP was lower compared to MaxEnt, as indicated by AUC, KAPPA, and TSS values, suggesting that MaxEnt performed excellently (Warren and Seifert, 2011; Zhu et al., 2012). Then, a detailed analysis on the suitable habitat of three *Fritillaria* species under current and future climate scenarios, and also a prediction of ecological niche overlapping was carried out. All the results would function as an important step in formulating a feasible strategy for their conservation and artificial cultivation.

Our model indicated that *the* center area for *F. cirrhosa* was in the south-west Sichuan province. This result was in agreement with the previous study that found this species mainly distributed in the Himalayan–Hengduan Mountain area (Li X. et al., 2017). Interestingly, some areas of Gansu, Shanxi, Henan, Guizhou, and Hubei provinces were also evaluated to be highly suitable habitats for this species, and this would provide areas for introducing *F. cirrhosa* in the future. Compared with *F. cirrhosa*, *F. unibracteata* and *F. przewalskii* were intensively distributed in Sichuan, Qinghai, Gansu, and Tibet, respectively.

Among the environmental variables, precipitation, elevation, and temperature contributed mostly to the distribution model for three *Fritillaria* species in respect to other variables, indicating that these factors played role in controlling their distribution. This result was supported by the fact that the climatic factors of an area acted as key elements for population regeneration. Most of the distribution area, including the center area, of three *Fritillaria* species received the influence of monsoon from the Indian and Pacific oceans and has sufficient precipitation from May to September. Water was reported to play an essential role in root growth (Sharma et al., 2021), seed dormancy and germination (Sheppard et al., 2014), and leaf growth and photosynthesis (Peng et al., 2021). Water deficiency would activate transcription factors and thus promote the expression of stress-related genes (Van Beek et al., 2021), whereas sufficient water would increase stomatal and leaf area (Mozo et al., 2021) and promote dormancy and germination (Chritine et al., 2014; Sheppard et al., 2014). In this study, three *Fritillaria* species shared similar precipitation, indicating the similar water requirement of the three species. As annual precipitation, lower than 335.98 mm, greatly reduced the probability of the presence of three *Fritillaria* species, it was considered to be an important constraint. Especially, the suitable precipitation in several growth stages of *Fritillaria* species was of importance. Especially, September to October was sowing time for *Fritillaria*, and thus the suitable precipitation in this period would play a significant role in its seed germination and next-step growth. Second, the moderate precipitation in May implied that the water requirement was necessary for the bulb filling of *Fritillaria* species. During the filling stage, it was reported that balanced water supplement improved not only the seed formation but also nutrient accumulation in seed and next-step yield, while unbalanced water supplement would promote plant diseases, such as *Fritillaria* rust and root rot from April to June. Meanwhile, previous studies have shown that freezing rain would cause plant cellular damage, pollen abortion, and declined height (Sheppard et al., 2014), and thus the low precipitation in autumn was beneficial to avoid the frost damage of *Fritillaria* species.

The altitude usually interacted with the temperature and light intensity, which had a significant influence on the accumulation of metabolites and nutrients in underground bulbs. Moreover, altitude was also correlated with UV-B, which showed influence on the subaerial organs of plants. However, little was known about the influence of UV-B radiation on the growth of *Fritillaria*, and quantitative research studies were further recommended. The similar high altitude of *F. cirrhosa* and *F. unibracteata* ensured both species to use the relatively low temperature to reduce the energy consumption of respiration, while relatively high light intensity could increase the photosynthesis, which was beneficial for the accumulation of metabolites and nutrients in underground bulbs. This result was in agreement with a previous report by Cunningham et al. (2018), who indicated that the occurrence of *F. cirrhosa* was in high altitude (from 2,700 to 4,600 m). Compared with those of *F. cirrhosa* and *F. unibracteata*, *F. przewalskii* displayed lower optimum altitude (2455.73 m), indicating that the growth of *F. przewalskii* was associated with relatively weaker light intensity, lower temperature, and UV-B.

Temperature is the third factor that was reported to affect the vegetative growth (Li et al., 2009) and bulbs dormancy (Marković et al., 2020) of *Fritillaria* species. The seeds of most *Fritillaria* species contained immature embryos, and thus dormancy breaking of seeds in *Fritillaria* species was typically complex, requiring after-ripening process (Chen et al., 1993). Treatment of *Fritillaria* seeds with cold stratification would break the dormancy seed and shorten germination time (Zhang, 1992). In addition, the relatively narrow Bio4 indicated that the survival of *Fritillaria* species might be sensitive to temperature change. This result was in agreement with a previous report by Li et al. (2019), who indicated that the alpine plants were more sensitive to temperature variability.

With greenhouse gas emissions, some species will migrate to high latitude or high elevation, while other species maintained the current location by changing physiologically or phenologically (Zhang et al., 2018; Faticov et al., 2021). Our prediction showed that the shift of *F. cirrhosa* from current altitude to higher altitude would become gradually more significant. Furthermore, the centroidal elevation of both *F. cirrhosa* and *F. przewalskii* shift were higher than the current habitat, which might be facilitated through adaptations. However, *F. unibracteata* might adapt to the changes of greenhouse gas emissions, as shown in unchanged centroid of suitable areas. Under higher concentrations of greenhouse gas emissions scenario, suitable habitat range of three species showed similar tendency to increase despite varying degrees. Under the highest emission (SSP585) scenario, the suitable area of *F. cirrhosa* and *F. przewalskii* reached the maximum 196.33 and 224.68%, respectively, in respect to that of the current situation, while those of *F. unibracteata* reached the maximum 172.65% under medium emission (SSP370) in respect to the current situation. That result was consistent with that of previous research studies, which indicated that temperature had a positive effect on plants by accelerating the process of phenology, improving growth rate and size, and increasing the soil nutrients (Jia et al., 2016; Li F. et al., 2017; Zettlemoyer et al., 2019). However, the sensitiveness of various species on temperature might be due to different adaptive rates, despite the adaptive mechanism being elusive.

Another consideration that would influence the distribution of species was the ecological niche. Such high Schoener’s *D* and Hellinger’s *I* values suggested that three *Fritillaria* species shared highly similar environmental factors and showed a high ecological niche. Consistently, three species shared commonly important environmental factors, including precipitation, elevation, and temperature, and corresponding similar optimum values. Moreover, as shown in Figure 7, the highly suitable habitats of three *Fritillaria* species were overlapped, and thus the current distributions of three species in this overlapping area could be estimated. As a result, approximately 50% distributions of *F. przewalskii* were located in the overlapping area, indicating the high ecological niche among three species. Generally, plants with close a relationship share a similar mechanism to adapt to living environments in the evolution process (Milanesi et al., 2018), and thus it was probable to find a correlation between phylogenetic relationships and ecological niche overlap among these species. To understand the correlation between ecological niche and phylogenies, the phylogenetic tree between *Fritillaria* species was constructed. In the phylogenetic tree, *F. cirrhosa*, *F. unibracteata*, and *F. przewalskii* formed a close clade with *Fritillaria unibracteata var wabuensis* and represented a close relationship. Therefore, the high accuracy supported that the spatial distribution was related to the phylogenies despite the relatively weak *P*-value (Table 2). The weak significance was also observed in *Pipridae* reported by Villegas et al. (2021) and *Profundulidae* reported by Miguel Calixto-Rojas et al. (2021). One possible reason is that the species evolution was controlled by multiple factors, such as spatial distribution, sexual reproduction, and geographical barrier (Zheng et al., 2021). Hopefully, the statistical value would be more reliable if another or more factors that contribute to species evolution are added in the correlation analysis. Another reason might be the limited species in this study. Tucker et al. (2018) showed that phylogenetic diversity correlated with functional diversity increasingly strongly as more species are included in the functional diversity measure. Hopefully, phylogenetic diversity might be a useful surrogate for high-dimensional trait diversity on the condition of sufficient *Fritillaria* samples. The third is the application of advanced machine learning that might provide a strategy; Grundler and Rabosky (2020) developed Dirichlet-multinomial framework to model resource use evolution on phylogenetic trees.

In addition, central Sichuan and the border regions between Sichuan, Qinghai, and Gansu Province were the main overlapping highly and moderately suitable areas of the three *Fritillaria* species. Meanwhile, the specific highly suitable habitat of *F. cirrhosa*, *F. unibracteata*, *F. przewalskii* were mainly distributed in southwest Sichuan, the border regions between Sichuan, Qinghai, and Gansu, respectively (Figure 7). For example, the non-overlap highly and moderately suitable habitats will be the preferable choices for cultivation to decrease habitat competition among species, improve production, and protect rare species as well.

## Conclusion

The role of future climate on the distribution of *Fritillaria* species is of vital importance for conservation. Our results indicated that the suitable habitat for three *Fritillaria* species tends to increase under the scenario for medium and highest concentration of greenhouse gas emissions. However, under the emission of greenhouse gas, we predicted that *F. cirrhosa* moved to high latitude and high elevation, while *F. przewalskii* migrated to high latitude and *F. unibracteata* did not migrate. The spatial and temporal shifts as well as the niche overlap between the three species would be a useful reference in developing a reasonable conservation strategy for those three medicinally important species.

## Data Availability Statement

The original contributions presented in the study are included in the article/Supplementary Material, further inquiries can be directed to the corresponding authors.

## Author Contributions

RJ and MZ performed the experiments. RJ, MZ, YQ, GT, SH, and HQ analyzed the data. JZ and HL contributed to the materials and analysis tool, wrote and were responsible for the manuscript. All authors contributed to the article and approved the submitted version.

## Conflict of Interest

The authors declare that the research was conducted in the absence of any commercial or financial relationships that could be construed as a potential conflict of interest.

## Publisher’s Note

All claims expressed in this article are solely those of the authors and do not necessarily represent those of their affiliated organizations, or those of the publisher, the editors and the reviewers. Any product that may be evaluated in this article, or claim that may be made by its manufacturer, is not guaranteed or endorsed by the publisher.

## Footnotes

1
https://www.cvh.ac.cn/

2
http://ppbc.iplant.cn/

3
http://www.gbif.org

4
https://map.bmcx.com
