# Supplementary material for: Modeling of the Potential Geographical Distribution of Three Fritillaria Species Under Climate Change
Source: Front Plant Sci. 2022 Jan 10;12:749838. doi: 10.3389/fpls.2021.749838 (PMC8784777; doi:10.3389/fpls.2021.749838)
Supplement: Supplementary file 4 [file Table_4.docx]

Supplementary Table 4 Evaluation of MaxEnt and GARP models at various training/testing sets.

| Species | MaxEnt | | | GARP | | |
| --- | --- | --- | --- | --- | --- | --- |
|  | 80/20 (training/testing) | 75/25 (training/testing) | 70/30 (training/testing) | 80/20 (training/testing) | 75/25 (training/testing) | 70/30 (training/testing) |
| *F. cirrhosa* | 0.966(0.004)/0.702/0.644 | 0.970(0.006)/0.704/0.703 | 0.969(0.006)/0.731/0.730 | 0.821(0.030)/0.654/0.641 | 0.5(0)/0/0 | 0.5(0)/0/0 |
| *F. unibracteata* | 0.973(0.010)/0.851/0.785 | 0.972(0.012)/0.913/0.890 | 0.972(0.006)/0.882/0.875 | 0.832(0.014)/0.679/0.665 | 0.823(0.040)/0.654/0.647 | 0.792(0.032)/0.605/0.584 |
| *F. przewalskii* | 0.980(0.004)/0.882/0.872 | 0.981(0.006)/0.855/0.913 | 0.984(0.005)/0.871/0.859 | 0.656(0.028)/0.391/0.312 | 0.686(0.081)/0.442/0.372 | 0.641(0.015)/0.355/0.282 |

Note: The AUC/KAPPA/TSS values were separated by slashes. The SD value was listed in the bracket.
