# Supplementary material for: Modeling of the Potential Geographical Distribution of Three Fritillaria Species Under Climate Change
Source: Front Plant Sci. 2022 Jan 10;12:749838. doi: 10.3389/fpls.2021.749838 (PMC8784777; doi:10.3389/fpls.2021.749838)
Supplement: Supplementary file 5 [file Table_5.docx]

Supplementary Table 5

Dynamics of changes in distribution area for three *Fritillaria* species under current and four future scenarios.

| Portion of area (% ) | *Fritillaria cirrhosa* | | | | *Fritillaria unibracteata* | | | | *Fritillaria przewalskii* | | | |
| --- | --- | --- | --- | --- | --- | --- | --- | --- | --- | --- | --- | --- |
|  | Not suitable | Low suitable | Moderate suitable | High suitable | Not suitable | Low suitable | Moderate suitable | High suitable | Not suitable | Low suitable | Moderate suitable | High suitable |
| Current | 92.90 | 5.44 | 1.43 | 0.24 | 94.98 | 3.68 | 1.09 | 0.25 | 96.36 | 2.44 | 1.00 | 0.20 |
| SSP126 2021-2040 | 91.22 | 6.41 | 1.99 | 0.38 | 93.64 | 4.69 | 1.33 | 0.32 | 94.05 | 3.73 | 1.64 | 0.57 |
| SSP126 2041-2060 | 90.52 | 6.67 | 2.32 | 0.48 | 93.44 | 4.81 | 1.40 | 0.34 | 95.08 | 3.16 | 1.36 | 0.39 |
| SSP126 2061-2080 | 92.29 | 5.35 | 1.89 | 0.46 | 93.12 | 4.85 | 1.66 | 0.36 | 93.52 | 4.17 | 1.66 | 0.65 |
| SSP126 2081-2100 | 91.60 | 5.95 | 2.06 | 0.38 | 93.80 | 4.50 | 1.39 | 0.30 | 94.66 | 3.56 | 1.38 | 0.4 |
| SSP245 2021-2040 | 90.41 | 7.18 | 1.99 | 0.41 | 93.68 | 4.40 | 1.56 | 0.36 | 93.81 | 3.84 | 1.84 | 0.49 |
| SSP245 2041-2060 | 90.58 | 6.85 | 2.11 | 0.45 | 94.15 | 4.10 | 1.29 | 0.46 | 94.81 | 3.49 | 1.29 | 0.40 |
| SSP245 2061-2080 | 88.43 | 7.74 | 3.07 | 0.75 | 93.76 | 4.31 | 1.53 | 0.40 | 93.45 | 4.01 | 1.84 | 0.70 |
| SSP245 2081-2100 | 86.76 | 8.14 | 4.12 | 0.97 | 92.12 | 5.86 | 1.61 | 0.41 | 92.62 | 4.25 | 2.24 | 0.89 |
| SSP370 2021-2040 | 87.83 | 8.62 | 2.95 | 0.58 | 94.07 | 4.20 | 1.39 | 0.34 | 94.69 | 3.34 | 1.54 | 0.43 |
| SSP370 2041-2060 | 89.29 | 7.34 | 2.59 | 0.77 | 93.97 | 4.45 | 1.29 | 0.28 | 95.68 | 2.87 | 1.17 | 0.26 |
| SSP370 2061-2080 | 86.50 | 8.08 | 4.01 | 1.39 | 91.34 | 6.24 | 1.96 | 0.46 | 93.67 | 3.67 | 1.82 | 0.84 |
| SSP370 2081-2100 | 87.66 | 8.16 | 3.24 | 0.93 | 91.73 | 5.82 | 1.91 | 0.54 | 93.29 | 3.98 | 2.00 | 0.74 |
| SSP585 2021-2040 | 87.83 | 8.62 | 2.95 | 0.58 | 93.56 | 4.73 | 1.35 | 0.36 | 94.41 | 3.56 | 1.54 | 0.48 |
| SSP585 2041-2060 | 90.45 | 7.01 | 2.03 | 0.49 | 93.54 | 4.62 | 1.50 | 0.34 | 95.35 | 3.05 | 1.29 | 0.29 |
| SSP585 2061-2080 | 86.82 | 8.07 | 3.95 | 1.15 | 93.25 | 4.71 | 1.71 | 0.33 | 93.14 | 3.87 | 2.07 | 0.92 |
| SSP585 2081-2100 | 86.04 | 8.52 | 3.90 | 1.53 | 92.01 | 5.55 | 2.08 | 0.36 | 91.83 | 4.64 | 2.43 | 1.10 |
